# Macrocognition through the Multiscale Enaction Model (MEM) Lens: Identification of a Blind Spot of Macrocognition Research

**DOI:** 10.3389/fpsyg.2016.01123

**Published:** 2016-07-27

**Authors:** Eric Laurent, Renzo Bianchi

**Affiliations:** ^1^Laboratory of Psychology (EA 3188), University Bourgogne Franche-ComtéBesançon, France; ^2^Institute of Work and Organizational Psychology, University of NeuchâtelNeuchâtel, Switzerland

**Keywords:** complexity, enaction, enactivism, motivated cognition, multiscale cognition, needs

“Given a dark room and a highly motivated subject, one has no difficulty in demonstrating Korte's Laws of phenomenal movement. Lead the subject from the dark room to the market place and then find out what it is he sees moving and under what conditions, and Korte's Laws, though still valid, describe the situation about as well as the Laws of Color Mixture describe one's feelings before an El Greco canvas.”

Bruner and Goodman (1947, p. 33)

## Introduction

Macrocognition research is concerned with cognitive processing in complex environments, goal-oriented action, goal combination and competition, cognitive-affective and cognitive-social interactions, distributed processing, and situatedness. These interests are critical to the theoretical modeling of cognitive systems for at least two reasons: (1) complexity is pervasive (and generally increases from laboratory to daily life situations), and (2) efforts are needed within (and across) all scientific fields to give meaning to, and a more global picture of, usually separate(d) knowledge fields.

In the present paper, and exactly for these two same reasons, we examine the status of “macrocognition” and suggest that *epistemologically*, “macrocognition” should not be regarded as different from other forms of cognition, including what has been called “microcognition” (Clark, 1989). Microcognition usually refers to more “internal,” “subpersonal” determinants of cognitive processing (e.g., neuronal activity involved in visual perception). However, in contrast to what is sometimes found in the macrocognition literature, we do not consider microcognition as a set of “invariant” processes or “building blocks” of cognition (Letsky and Warner, 2008, p. 9). Rather, we propose here that complexity and dynamics characterize both macrocognition and microcognition. Moreover, macrocognition cannot “shunt” microcognition. Rather than promoting a new functionalism at the macroscale, we recommend that a more unitary, multiscale^1^ approach to cognition be developed. Human cognition is complex and distributed, as is the biological network on which it relies. We suggest studying the generic properties of cognition through flexible analysis scales rather than creating specific fields or categories of cognition as a function of the scale of interest. In the following lines, we rely on a multiscale model of perception-action cycles' emergence, the Multiscale Enaction Model (MEM; Laurent, 2014), in which context is conceived of as being both multiple and multiscale. First, this model allows us to consider multiple interactions between processes, in line with macrocognition research's aims. Second, it highlights the need to flexibly conceive cognitive interactions at multiple scales and to reunite cognition and aims, including basic, embodied physiological goals (e.g., hydration, energy recompletion), which do not need to be consciously elaborated.

## Why cognition can be “macrocognition”

The term “macrocognition” can refer to at least two perspectives over cognition. The first perspective characterizes augmented cognition theories and stresses the role of informational complexity and distributed or extended cognition. It can be opposed to more elementary views on information processing and to analytical research strategies. Macroscale factors (e.g., socioeconomical position of a family) can change microcognition (e.g., object size estimation) even in laboratory settings (Bruner and Goodman, 1947). This point is important for later discussion presented in our paper, because microcognition can neither be viewed as isolated from large-scale influences (limits of experimental-analytic approaches to cognition) nor be considered as fixed or as a set of “invariable building blocks” of cognition (limits of some macrocognition approaches, discussed later). Therefore, cognition rather appears to be enacted through interactions relating microscopic and macroscopic levels, such that a rupture between micro and macroscale analyses does not seem to be *epistemologically* sound.

The second perspective is related to the nature of the cognitive determinants that are valued, with the prefix “macro” referring to relatively large-scale influences (e.g., cognitive-social interactions), as opposed to more regional mutual influences (e.g., neuro–neuronal interactions). In this perspective “macrocognition” is often seen as being more “ecologically valid,” or as enhancing “external validity” because it focuses on wide-range interactions that can be encountered in daily situations:

“*Macrocognition is a term coined by Pietro Cacciabue and Erik Hollnagel to indicate a level of description of cognitive functions that are performed in natural (versus artificial laboratory) decision-making settings […] the methodology for macrocognition focuses on the world outside the lab. This includes contexts designated by such terms as the ‘field setting’, the ‘natural laboratory’, and the ‘real world.”’*

(Klein et al., 2003, p. 81)

We are sympathetic with the view that cognition is embedded in a network of contextual influences (i.e., the first perspective), but we anticipate limitations to the second view, which may imply a new reductionist functionalism—a large-scale equivalent to functionalist views over microcognition. Indeed, *there is no pre-set, well-suited scale of analysis*. As complexity is pervasive and multiscale, the scale at which processes should be described has to be *flexible* rather than fixed.

## Why macrocognition cannot shunt microcognition: from exogenous to endogenous complexity

The term “macrocognition” usefully highlights the need for a larger scope of analysis than the one characterizing most laboratory-based experiments. However, what is usually thought of as an “external” or “environmental” factor actually combines with the organism state so that one cannot exclude any term of the interaction at any single moment. The activity of any part of an organism depends on the activity of the other parts to which it is linked. For instance, even when social-environmental complexity related to the task at hand is high (e.g., real-world lottery), factors affecting low-level biological parameters (such as ambient temperature, Cheema and Patrick, 2012) can have impacts over cognition (e.g., consumer choice). The internal resource dynamics (e.g., related to hydration) changes the willingness to make difficult gambles. Furthermore, a great amount of social psychology research, which is supposed to capture *social* complexity, is grounded in self-reported measures and individual interpretation of external complexity. In order to produce self-reports, internal construction of what is reported is a *prerequisite to* data communication and processing; this internal construction involves microscale activity (e.g., at the cellular level).

There is no macroscopic-level influence on behavior without (a) prior biological or psychological integration of the values associated with the factors of influence and (b) competition between and/or combination with the current goals and needs of the organism. Failing to recognize the complex nature of the phenomena constituting a human being can give rise to reductionism, be at the microscale or at the macroscale level. From this standpoint, suprapersonal (e.g., social), personal, and subpersonal (e.g., cellular), levels of analysis should meet. The terms “suprapersonal” and “subpersonal” refer to different scale levels in the analysis of cognition but do not imply an opposition between complexity and simplicity. Suprapersonal factors (e.g., social influences) are currently more easily detectable from a macroscale level of analysis whereas “subpersonal” factors (e.g., genetic influences) are currently more easily observable from a microscale level of analysis. However, considering one as being complex and the other one as being elementary *and* invariant would be misleading. For instance, one cannot pretend that “genetic” determinants of cognition do not involve a wealth of interacting mechanisms that influence each other (see Flint, 1999; Hill et al., 2014). In the following lines, we suggest that macrocognition and microcognition should be conceived within a single epistemological framework.

## Why macrocognition does not *epistemologically* differ from microcognition in the multiscale enaction model

Enactive systems produce information and knowledge by acting in their environment. In MEM (Laurent, 2014), each cell is conceived as an autopoietic structure^2^ which tends to optimize its own functioning by interacting with other cells or groups of cells. Perception-action cycles in MEM rely on those interactions because what is searched for in the environment depends on internal needs and goals. Internal needs and goals can be described at different scales. Any “external” or “ecological” influence over behavior is a *transaction* between embodied personal history (i.e., the current mode of coupling between the organism and its environment, subsequent to previous evolution and learning), goals, needs or orientations and external stimulation. Put differently, macrocognition cannot be correctly thought of without describing the interactions between the current biological state and motivation of the organism on the one hand and macroscale stimulation on the other hand.

Distributed cognition is pervasive, not only at the subpersonal level, but also at the suprapersonal level (e.g., networks of interacting individuals). There should be no epistemological rupture in the conception of distributed cognition, at physical, biological, and psychological levels. Huebner (2014) reviewed many studies suggesting that collective performance strongly depends on the coordinative properties of couples or groups, such that the collective performance cannot be reduced to the sum of individual performances. Interestingly, cognitive distribution and coordinative patterns are fundamental emerging features of groups of cells within neural networks (Craddock et al., 2013), brain areas (Bressler and Menon, 2010), and human groups (Goldstone et al., 2008). At any level, the distribution of cognition allows for the sharing of the informational load the organism is dealing with and the generation of new information through exchanges between the organism's parts. In MEM, a multiscale unifying principle is hypothesized within the central nervous system, which relates external and internal events to the organism's goals, such that both macro and microscale influences combine and are weighted as a function of their value for the organism. In MEM, the interactions between needs and goals (considered from the cellular to the psychosocial and economic levels^3^) and perception-action cycles are basic foundations for resource allocation given the limitations in time and processing power. According to the model, teleological^4^ dimensions of activity arise from the combination of need expression at the cellular and the cell network levels, and spread out to the organism and phenomenological experience through diffusion, competition, and cooperation. In this conception, the goal-directed nature of cognition makes it critical to capture any kind of influence that can modify the organism's goals. In this sense, any macroscopic-level factor should be put in the context of the organism state, as—in the other way round—the organism's informational processing and behavior should be considered in the context of larger environmental influences. In other words, in a radically distributed cognitive framework, *distribution has no pre-set scale of analysis*. Rather, distribution should be considered in every network that allows for information exchange and influences need/goal/aim satisfaction or frustration, be at cellular, cognitive, or social-affective levels. By relating micro *and* macroscale information integration to internal goals and needs, this multiscale approach provides us with tools to reunite macro and microscopic processes and levels of analysis.

## Scale flexibility in distributed cognition research: ending up with the blind spot of “macrocognition research”

“*What is a thing at one level may be relations among (different) things at another*.”

(Kelso, 1995, p. 97)

Though we subscribe to the macrocognition perspective for its emphasis on complexity, we warn the reader against the risks associated with a fixed-scale approach to cognition. Because macrocognition researchers stress the role of complexity, they should develop scale flexibility in their analyses. Even what is referred to as “macrocognition” by some researchers working on emotional context of behavior is identified as microscopic by others working on social networks. This does not change anything to the fact that, in order to analyze complex behaviors, we need to contextualize them. As a function of the scale of analysis, what can be considered a “context” varies.

Arguably what should be regarded as “ecologically valid” is the capture of multiscale interactions in experimental—or, more largely, empirical—settings that are found in everyday situations (rather than simply macroscale interactions). On those bases, and following what we discussed earlier, neglecting microscopic factors may be as harmful as neglecting macroscopic factors. In any instance of fixed-scale analysis, cognition is most probably regarded as a set of “functions” that process information under the influence of a limited number of “causes.”

We invite the reader to pay attention to a blind spot that we have identified in the literature on macrocognition. The “macrocognition research blind spot” consists in associating “emergence,” “dynamics” and “complexity” with macrocognition as opposed to “invariant processes” or “building blocks” of cognition, which would be identified by microcognition research (Klein et al., 2003). We consider this distinction misleading. As discussed earlier in this paper, microcognition is also emergent, complex, and dynamic (Laurent, 2014). Distinguishing micro from macrocognition research on the basis of emergence, complexity, and dynamics (or “reality”) is neither empirically nor theoretically or logically founded. The problems associated with mainstream cognitive psychology/science (e.g., poor consideration for emergence, analytic approaches, lack of dynamic frameworks) should not be confused with the issue of the scale (i.e., micro, macro) at which the analysis is performed.

Relatedly, we do not adhere to the recurrent statements (or judgements) found in the Macrocognition literature on what “reality” is:

“*Microcognition relinquishes the coupling between the phenomenon and the real context to the advantage of the coupling with the underlying theory or model.”*

(Cacciabue and Hollnagel, 1995, p. 57)

We rather call for a true *contextual relativism* where factors such as hydration level, laboratory settings, “internal” biological disorders, or mood fluctuations are as real as (i) the biomechanical constraints, goals, prescriptions, machines, pervasive information systems, and social context surrounding task realization and (ii) the parameters to be coordinated, which participate in emerging cognition and behaviors.

If macrocognition is to become a reference framework for the cognitive science of embedded agents, then the contexts under scrutiny should be flexibly defined, and their role theoretically reconstructed and empirically tested.

We hope that researchers interested in complexity will not add a new scale to functionalism. In other words, macrocognition should not exclude microcognition. As put by Minsky (1988), “*each higher level of description must* add *to our knowledge about lower levels, rather than replace it”* (p. 26). We note that this addition of knowledge should not be merely scale-specific. Rather, it should involve working on the interactions between different scales and reporting what identifies/differentiates distributed cognition at different scales. This is a basic condition to approach behavioral complexity and to develop more unitary frameworks in psychology and life sciences.

## Author contributions

EL wrote the initial draft of the manuscript. EL and RB contributed to review several versions of the manuscript and have approved the final manuscript.

### Conflict of interest statement

The authors declare that the research was conducted in the absence of any commercial or financial relationships that could be construed as a potential conflict of interest.

## Footnotes

^1^By using the term “multiscale,” we refer to *multiple levels of observation and analysis* (e.g., cellular, individual, social).

^2^The Autopoiesis refers to self-production and maintenance of a “systemic variable”; an autopoietic system is a “homeostat” in which “the critical variable is *the system's own organization”* (Stafford Beer, *Preface of* Autopoiesis, *The Organization of the Living*, In Maturana and Varela, 1980, p. 66).
“*An autopoietic machine is a machine organized (defined as a unity) as a network of processes of production (transformation and destruction) of components that produces the components which: (i) through their interactions and transformations continuously regenerate and realize the network of processes (relations) that produced them; and (ii) constitute it (the machine) as a concrete unity in the space in which they (the components) exist by specifying the topological domain of its realization as such a network.”* (Maturana and Varela, 1980, pp. 78–79)

^3^Even if our view may be different from Maturana's regarding goals and needs, we completely agree with him when he considers that a similar organization can be found in many different structures: “any given organization may be realized through many different structures, and […] different subsets or relations included in the structure of a given entity, may be abstracted by an observer […] as organizations that define different classes of composite unities (Maturana, 1980, p. XX). For more information about biocomputational bases for goal and need summations, see Laurent (2014).

^4^As used here, the term “teleological” does not refer to any form of metaphysical finalism.
